# Adjunctive corticosteroids for *Pneumocystis jiroveci *pneumonia in patients with HIV infection: a meta-analysis of randomised controlled trials

**DOI:** 10.1186/1471-2334-5-101

**Published:** 2005-11-07

**Authors:** Matthias Briel, Remy Boscacci, Hansjakob Furrer, Heiner C Bucher

**Affiliations:** 1 Institut für klinische Epidemiologie, Universitätsspital Basel, 4031 Basel, Switzerland; 2 Klinik und Poliklinik für Infektiologie, Inselspital, 3010 Bern, Switzerland; 3 Klinik für Infektiologie, Universitätsspital Basel, 4031 Basel, Switzerland

## Abstract

**Background:**

The objective of this study was to review the effects of adjunctive corticosteroids on overall mortality and the need for mechanical ventilation in HIV-infected patients with *Pneumocystis jiroveci *pneumonia (PCP) and substantial hypoxemia (arterial oxygen partial pressure <70 mmHg or alveolar-arterial gradient >35 mmHg on room air).

**Methods:**

We conducted a systematic search of the literature for randomised trials published up to December 2004. Selected trials compared adjunctive corticosteroids with placebo or usual care in HIV-infected patients with PCP and reported mortality data. Two teams of reviewers independently evaluated the methodology and extracted data from each primary study.

**Results:**

Six studies were included in the meta-analysis. Risk ratios for overall mortality for adjunctive corticosteroids were 0.54 (95% confidence interval [CI], 0.38–0.79) at 1 month and 0.67 (95% CI, 0.49–0.93) at 3–4 months of follow-up. Numbers needed to treat, to prevent 1 death, are 9 patients in a setting without highly active antiretroviral therapy (HAART) available and 22 patients with HAART available. Only the 3 largest trials provided data on the need for mechanical ventilation with a risk ratio of 0.37 (95% CI, 0.20–0.70) in favour of adjunctive corticosteroids.

**Conclusion:**

The number and size of trials investigating adjunctive corticosteroids for HIV-infected patients with PCP is small, but our results suggest a beneficial effect for patients with substantial hypoxemia.

## Background

With the introduction of highly active antiretroviral therapy (HAART) more than a decade ago, the incidence of *Pneumocystis jiroveci *pneumonia (PCP) [1] has decreased significantly in the Western hemisphere. However, PCP still remains the most common opportunistic infection in patients infected with the human immunodeficiency virus (HIV) [2]. Among patients with HIV infection and PCP the mortality rate is 10 to 20% during the initial infection and increases substantially with the need for mechanical ventilation [3]. In 1990 an expert panel recommended the use of corticosteroids for HIV-infected patients with PCP and substantial hypoxemia (initial arterial oxygen partial pressure of <70 mmHg or alveolar-arterial gradient >35 mmHg on room air) based on the evidence from five randomised controlled trials [4]. This consensus statement still represents the basis of current treatment guidelines [5]. However, at the time this statement was made, one trial was not yet completed [6], two trials were stopped prematurely [7,8], and one trial was not published in full [9]. In 1992 a systematic review qualitatively summarised the same incomplete data [10].

We present an updated systematic review and meta-analysis of randomised controlled trials to assess the magnitude of effects of adjunctive corticosteroid therapy on overall mortality and the need for mechanical ventilation in HIV-related PCP. In addition, we provide numbers needed to treat that may serve as estimates for the expected benefit of adjunctive corticosteroid therapy in the presence and absence of HAART.

## Methods

### Search for relevant studies

We searched MEDLINE (January 1985 – December 2004), EMBASE (January 1985 – December 2004) and the Cochrane Library (issue 4, 2004) without language restrictions to identify randomised controlled trials that compared adjunctive corticosteroids to control in HIV-infected patients with PCP. We used the terms steroid*, corticosteroid*, glucocorticoid*, pneumocystis, PCP, *carinii, *jiroveci as text words and *Glucocorticoids, Adrenal Cortex Hormones, Steroids, Pneumocystis Infections, Pneumocystis jiroveci*, and *Pneumonia, Pneumocystis *as Medical Subject Headings. We restricted the search to articles indexed as randomised controlled trials (publication type) or drug therapy (subject heading) or those that included the words random* or *placebo *in their titles or abstracts. We further reviewed the reference lists from previously published overviews [4,10], we searched UptoDate version 2005 and Clinical Evidence Concise (issue 12, 2004), contacted experts of the field, and searched reference lists of identified publications for citations of additional relevant articles.

### Study selection and data abstraction

Trials were considered eligible for this meta-analysis if they compared corticosteroids to placebo or usual care in HIV-infected patients with PCP in addition to baseline treatment with trimethoprim-sulfamethoxazole, pentamidine or dapsone-trimethoprim, used random allocation, and reported mortality data. We excluded trials in patients with no or mild hypoxemia (arterial oxygen partial pressure >70 mmHg or an alveolar-arterial gradient <35 mmHg on room air) and trials with a follow-up of less than 30 days.

Two teams of investigators (MB/HCB and RB/HF) assessed study eligibility and quality blinded to one another's rating and resolved any disagreement by consensus. Data of eligible trials were abstracted in duplicate. We assessed the quality of included trials with respect to concealment of treatment allocation; blinding of patients, caregivers or assessors of clinical outcomes; performance of a sample size calculation; and if the trial was stopped early [11]. The main endpoint for benefit of adjunctive corticosteroid therapy was overall mortality. A secondary endpoint was the need for mechanical ventilation.

### Statistical analysis

All analysis was according to the intent-to-treat principle. We pooled treatment effects across studies and calculated a weighted average risk ratio of overall mortality in the treatment and control groups by using a random effects model. We investigated the presence of publication bias by means of funnel plots [12]. We tested for heterogeneity with the Cochran Q test and measured inconsistency (I^2^; the percentage of total variance across studies that is due to heterogeneity rather than chance) of treatment effects across studies [13,14]. We carried out sensitivity analyses to examine treatment effects according to quality components of trials, and if publication was as a peer-reviewed article or just in abstract form. Numbers needed to treat were calculated by multiplying the mean relative risk reduction with an initial mean baseline risk [15]. All statistical analyses were performed using Stata 8.2 (StataCorp, College Station, Tex).

## Results

**Figure 1:**
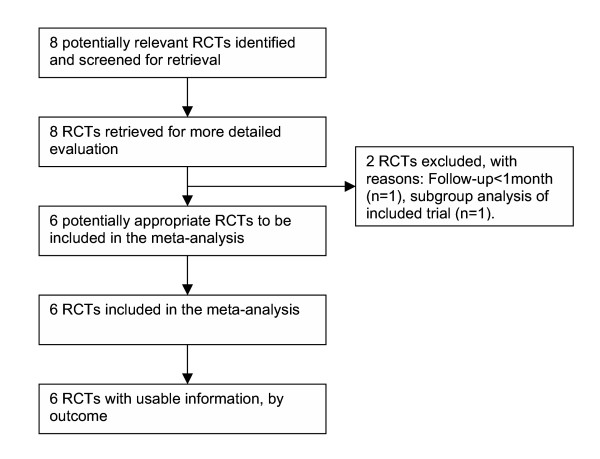
Flow diagram of trials. RCT, randomised controlled trial.

**Figure 2:**
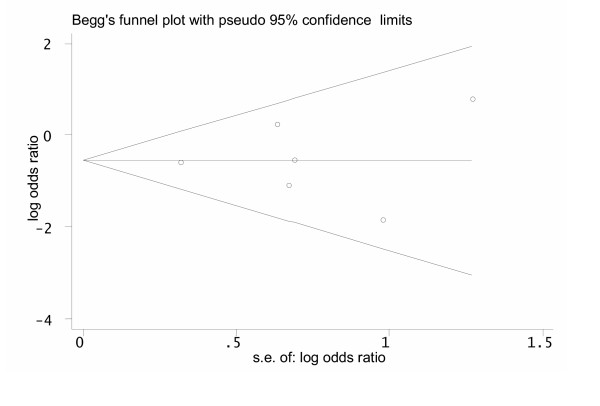
Funnel plot to evaluate the presence of a publication bias in trials investigating adjunctive corticosteroids for pneumocystis jiroveci pneumonia in HIV-infected patients. The funnel graph plots the log of the treatment odds ratio against the standard error (s.e.) of the log odds ratio (an indicator of sample size). Open circles represent trials included in the meta-analysis. The line in the centre indicates the summary log odds ratio. In the absence of a publication bias, the log odds ratio estimates from smaller trials are expected to be scattered above and below the summary estimate, producing a symmetric triangular or funnel shape. When smaller trials with larger log odds ratios are missing, the funnel plot appears asymmetric and may indicate the presence of a publication bias. In our systematic review the funnel plot looks symmetric. The Egger test for publication bias was not statistically significant (*P *= 0.91).

We identified 8 trials [6-9,16-19] that met our inclusion criteria (Figure 1). We excluded one trial [17] because it investigated only patients with mild hypoxemia and had a very short follow-up of only 3 days, and another trial [18] which turned out to be a subgroup analysis of a larger included trial [8] [see Additional file 1]. In total, there were 242 individuals in the intervention and 247 individuals in the control groups. The Funnel plot indicated no evidence for a publication bias (Figure 2). Characteristics of included trials are provided in Table 1.

**Table 1:** Characteristics of included trials

**Author (year) reference**	**Diagnosis of PCP**	**Baseline treatment for PCP**	**Oxygenation entry criteria**	**Corticoid (route)/initial daily dose/duration (days)**	Interval (max.) *	**Blinding patients/care givers/assessors**	**Concealed allocation/n centres**	**Sample size calculation/stopped early**	**Randomised individuals to I/C**	**Total deaths in I/C**
Clement et al. (1989) [9]	BAL, sputum	88% TMP-SMX, 12% Pentamidine	PaO_2_<51 mmHg (room air)	Methylprednisolon (IV)/240 mg/8 d	Unlimited	Yes/Yes/Unclear	Unclear/1	Unclear/No	19/22	9/9 at 56 days
Montaner et al. (1990) [8]	BAL	TMP-SMX, Pentamidine, Dapsone-TMP	85–90% O_2_-Saturation †	Prednisone (oral)/60 mg/7 d with 14 d tapering	48 h	Yes/Yes/Unclear	Yes/1	Yes/Yes	18/19	1/0 at 30 days 2/1 at 90 days
Bozzette et al. (1990) [16]	75% BAL, 15% sputum + presumed	80% TMP-SMX, 18% Pentamidine, 2% Dapsone-TMP	Hypoxemia ratio >75 ‡	Prednisone (oral)/80 mg/21 d or as baseline treatment	36 h	No/No/Unclear	Yes/6	Yes/No	123/128	13/28 at 31 days 20/33 at 84 days
Gagnon et al. (1990) [7]	BAL, biopsy, sputum	TMP-SMX	PaO_2_<75 mmHg (35% oxygen)	Methylprednisolon (IV)/160 mg/7–10 d	72 h	Yes/Yes/Unclear	Unclear/1	Yes/Yes	12/11	3/9 at 28 days 5/9 at 120 days
Nielsen et al. (1992) [6]	BAL, biopsy	TMP-SMX	PaO_2_<67.5 mmHg (room air)	Methylprednisolon (IV)/2 mg/kg/10 d	24 h	No/No/Unclear	Unclear/3	Unclear/Yes	30/29	2/9 at 34 days 4/9 at 90 days
Walmsley et al. (1995) [19]	BAL, biopsy, sputum	82% TMP-SMX, 17% Pentamidine, 1% Dapsone-TMP	PaO_2_<70 mmHg (room air) §	Methylprednisolon (IV)/80 mg/10 d	24 h	Yes/Yes/Unclear	Yes/3	Yes/No	40/38	4/6 at 35 days

Abbreviations: PCP, *Pneumocystis jiroveci *pneumonia; n, number; I/C, intervention/control group; BAL, bronchoalveolar lavage; TMP, trimethoprim; SMX, sulfamethoxazole; IV, intravenous.

* Maximal interval between initiation of baseline treatment for *Pneumocystis jiroveci *pneumonia and initiation of corticosteroid.

† Or 5% decrease on exercise.

‡ PaO_2 _divided by fraction of inspired oxygen.

§ Or alveolar-arterial oxygen gradient >40 mmHg if arterial blood gases could not be assessed on room air.

### Overall mortality

**Figure 3:**
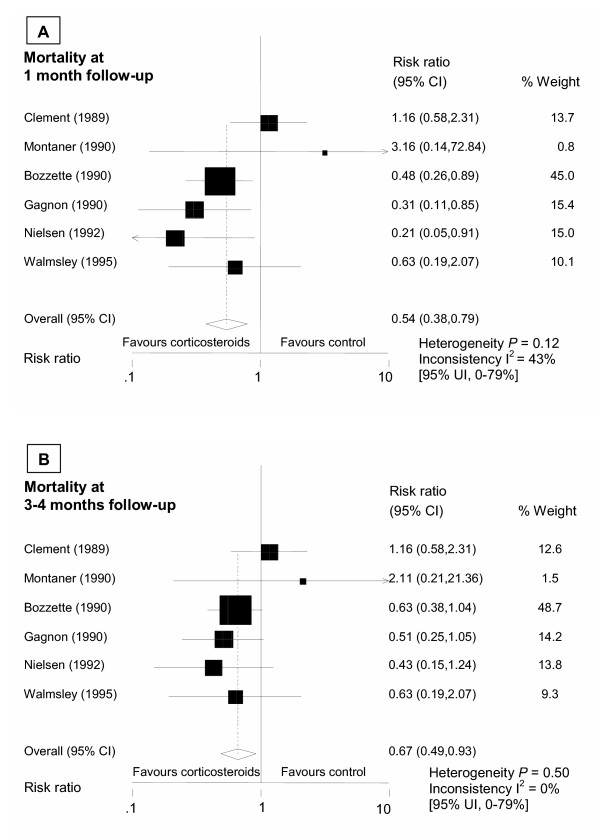
Summary estimates for overall mortality at 1 month (A) and 3–4 months (B) follow-up. The Cochran Q test for heterogeneity. I^2 ^as a measure of inconsistency (in percent). CI indicates confidence interval; UI, uncertainty interval.

Risk ratios for overall mortality were significantly reduced for adjunctive corticoids at 1 month (0.54;95% CI, 0.38–0.79) and at 3–4 months (0.67;95% CI, 0.49–0.93) of follow-up (Figure 3). We found some evidence for heterogeneity among trials at 1 month (test of heterogeneity, *P *= 0.12; I^2 ^= 43% [95% uncertainty interval [UI], 0%–78%]) whereas at 3–4 months treatment effects looked more homogenous (*P *= 0.46; I^2 ^= 0% [95% UI, 0%–75%]. In a sensitivity analysis heterogeneity was considerably reduced when the analysis was limited to trials with early (<3 days) adjunctive corticosteroids that were published in full, i.e. excluding Clement et al. [9] (summary risk ratio for mortality at 1 month: 0.45 (95% CI, 0.29–0.70), heterogeneity *P *= 0.49; I^2 ^= 0% [95% UI, 0%–79%]). In further sensitivity analyses for the mortality endpoint at 1 month summary risk ratios were 0.55 (95% CI, 0.32–0.93) in trials that reported concealed allocation [8,16,19], 0.74(95% CI, 0.45–1.21) in trials reporting blinding of patients and care-givers [7-9,19], and 0.64 (95% CI, 0.42–0.98) in trials not prematurely halted [9,16,19].

### Need for mechanical ventilation

Reliable data on the need for mechanical ventilation was only available for the 3 largest trials [6,16,19]. Again, the risk ratio for this endpoint was largely reduced in the group with early adjunctive corticosteroids (0.37;95% CI, 0.20–0.70; *P *= 0.40; I^2 ^= 0% [95% UI, 0%–90%]).

## Discussion

This systematic review of 6 randomised controlled trials in HIV-infected patients with PCP and substantial hypoxemia found a significant relative risk reduction of death for adjunctive corticosteroids of 46% at 1 month and of 33% at3–4 months. The average-weighted mean mortality in control groups of included trials at one month was 25%. This initial mortality-rate of 25% can be assumed in settings where HAART is not available which is still the case for most developing countries [20]. In this situation we estimated that 9 (95% CI, 6–19) HIV-infected patients with PCP have to be treated early with adjunctive corticosteroids to prevent 1 death during the first month after PCP diagnosis. In Western countries, where HAART is widely available, the respective number to treat was estimated to be 22 (95% CI, 16–48) patients assuming an initial mortality rate of 10% [21]. With regard to the need for mechanical ventilation the risk reduction for adjunctive corticosteroids was even greater in the investigated patient population, but the number of trials was small (n = 3).

Our study has several strengths and limitations. We conducted an extensive literature search to retrieve all eligible trials. However, formal testing for publication bias was not very powerful because of a relatively small number of included trials. Even with a symmetric looking funnel plot, such bias cannot be ruled out. Moreover, with a small number of included trials the uncertainty interval for the inconsistency among trials may not be very informative [13]. We focused mainly on mortality data that may be less prone to ascertainment bias, and we analysed the data according to the intent-to-treat principle to get more conservative estimates. Finally, the trials included in this meta-analysis used different corticosteroid regimen. So far, neither the dosing nor the length and tapering schedule of corticosteroids has been addressed adequately in randomised trials. In current recommendations [5] the corticosteroid schedule of the largest trial [16] was adapted.

There has been some concern among physicians treating patients with AIDS that further immunosuppression due to corticosteroid therapy could accelerate the onset of other HIV-related opportunistic complications [22,23]. However, with the exception of an increase in muco-cutaneous herpes simplex infection episodes [16], adjunctive corticosteroids were not associated with an increase in opportunistic complications in any of the included trials. A large cohort study which used a standard 21-day tapering course of adjunctive corticosteroids found no difference in the risk of AIDS-related complications apart from an increase in esophageal candidiasis [24].

It is possible that adjunctive corticosteroids are also beneficial for HIV-infected patients with mild hypoxemia due to PCP [17]. However, in this situation the short term mortality is low and possible unfavourable effects of corticosteroids might outweigh the benefits. Moreover, corticosteroids might also be beneficial for non-HIV-infected patients with severe PCP [25], but evidence from randomised controlled trials is still lacking.

## Conclusion

This meta-analysis confirmed and quantified the benefit of adjunctive corticosteroid therapy in HIV-infected patients with moderate-severe PCP. We estimated a relative risk reduction for overall mortality of 46% at 1 month and 33% at 3–4 months. We calculated numbers needed to treat of 9 patients for settings without HAART, and 22 patients with HAART available to prevent 1 death. Our results underline the conclusions of the early released consensus statement [4], and support current recommendations for the management of PCP in HIV-infected patients [5].

## Competing interests

The author(s) declare that they have no competing interests.

## Authors' contributions

MB and HCB conceived of the study and performed the literature search. MB, HCB, RB and HF checked eligibility and quality of trials, and extracted the necessary data. MB performed the statistical analyses and drafted the manuscript with the help of HCB, RB and HF. All authors read and approved the final version.

## Pre-publication history

The pre-publication history for this paper can be accessed here:



## Supplementary Material

Additional File 1 Table with characteristics of excluded trials and reasons for exclusion. Click here for file
