# Supplementary material for: Adjunctive corticosteroids for Pneumocystis jiroveci pneumonia in patients with HIV infection: a meta-analysis of randomised controlled trials
Source: BMC Infect Dis. 2005 Nov 7;5:101. doi: 10.1186/1471-2334-5-101 (PMC1309617; doi:10.1186/1471-2334-5-101)
Supplement: Additional File 1 — Table with characteristics of excluded trials and reasons for exclusion. [file 1471-2334-5-101-S1.doc]

### Table Excluded trials

| **Author of RCT (year) reference** | **Diagnosis of PCP** | **Baseline treatment for PCP** | **Oxygenation entry criteria** | **Corticoid (route)/ initial daily dose/ duration (days)** | **Interval (max.) *** | **Randomised patients to I/C** | **Reasons for exclusion** |
| --- | --- | --- | --- | --- | --- | --- | --- |
| Montaner et al. (1993) [18] | BAL | TMP-SMX, Pentamidine, Dapsone-TMP | >90% O_2_-Saturation at baseline, but ≥5% decrease on exercise. | Prednisone (oral)/ 60mg/ 7d with 14d tapering | 48h | 11/12 | **Subgroup-analysis of Montaner et al. (1990)** |
| Jeantils et al. (1993) [17] | Clinical suspicion | TMP-SMX | PaO_2_>70mmHg (room air) | Methylprednisolon (IV)/ 240mg/ 6d | 24h | 10 (total) | **Follow-up only 3 days, and PaO_2_ >70 mmHg on room air** |

Abbreviations: RCT, randomized controlled trial; PCP, *pneumocystis jiroveci* pneumonia; I/C, intervention / control group; BAL, bronchoalveolar lavage; TMP, trimethoprim; SMX, sulfamethoxazole; PaO_2_, arterial oxygen partial pressure; IV, intravenous.

* Maximal interval between initiation of baseline treatment for *pneumocystis jiroveci* pneumonia and initiation of corticosteroid.
